# Pathogenicity and transmission of a swine influenza A(H6N6) virus

**DOI:** 10.1038/emi.2017.3

**Published:** 2017-04-12

**Authors:** Hailiang Sun, Bryan S Kaplan, Minhui Guan, Guihong Zhang, Jianqiang Ye, Li-Ping Long, Sherry Blackmon, Chun-Kai Yang, Meng-Jung Chiang, Hang Xie, Nan Zhao, Jim Cooley, David F Smith, Ming Liao, Carol Cardona, Lei Li, George Peng Wang, Richard Webby, Xiu-Feng Wan

**Affiliations:** 1 Department of Basic Sciences, College of Veterinary Medicine, Mississippi State University, Mississippi State, MS 39759, USA; 2 Department of Infectious Diseases, St Jude Children's Research Hospital, Memphis, TN 38105, USA; 3 College of Veterinary Medicine, South China Agricultural University, Guangzhou 510462, Guangdong, China; 4 Laboratory of Respiratory Viral Diseases, Division of Viral Products, Office of Vaccines Research and Review, Center for Biologics Evaluation and Research, United States Food and Drug Administration, Silver Spring, MD 20993, USA; 5 Department of Population Medicine and Pathobiology, College of Veterinary Medicine, Mississippi State University, Mississippi State, MS 39759, USA; 6 Department of Biochemistry, Glycomics Center, School of Medicine, Emory University, Atlanta 30322, GA, USA; 7 College of Veterinary Medicine, University of Minnesota, St Paul, MN 30322, USA; 8 Department of Chemistry, Georgia State University, Atlanta, GA 30302, USA

**Keywords:** 2009 H1N1 influenza virus, aerosol transmission, glycan microarray, influenza A(H1N1)pdm09 virus, receptor binding, risk assessment, subtype H6N6 influenza virus, swine influenza virus

## Abstract

Subtype H6 influenza A viruses (IAVs) are commonly detected in wild birds and domestic poultry and can infect humans. In 2010, a H6N6 virus emerged in southern China, and since then, it has caused sporadic infections among swine. We show that this virus binds to α2,6-linked and α2,3-linked sialic acids. Mutations at residues 222 (alanine to valine) and 228 (glycine to serine) of the virus hemagglutinin (HA) affected its receptor-binding properties. Experiments showed that the virus has limited transmissibility between ferrets through direct contact or through inhalation of infectious aerosolized droplets. The internal genes of the influenza A(H1N1)pdm09 virus, which is prevalent in swine worldwide, increases the replication efficiency of H6N6 IAV in the lower respiratory tract of ferrets but not its transmissibility between ferrets. These findings suggest H6N6 swine IAV (SIV) currently poses a moderate risk to public health, but its evolution and spread should be closely monitored.

## INTRODUCTION

Influenza A virus (IAV) is an enveloped, segmented, single- and negative-stranded RNA virus belonging to the family *Orthomyxoviridae*. Migratory waterfowl are the natural reservoirs for IAVs, but these viruses also infect humans, domestic poultry, wild birds, pigs, dogs, cats, horses, mink and marine mammals, including seals and whales^1^ Human IAVs bind preferentially to *N*-acetylneuraminic acid-α2, 6-linked galactose (Neu5Acα2,6-Gal) receptors, whereas avian influenza viruses (AIVs) prefer *N*-acetylneuraminic acid-α2,3-linked galactose (Neu5Acα2,3-Gal) receptors.^2, 3, 4^ Swine are considered a ‘mixing vessel' of IAVs because they have both Neu5Acα2, 3-Gal and Neu5Acα2,6-Gal receptors throughout their respiratory tract. With these receptors, swine facilitate the generation of novel influenza reassortants and enable avian-like IAVs to obtain the ability to bind to human receptors,^5^ as has been hypothesized to have occurred during the genesis of viruses that caused the 1957 H2N2 and 1968 H3N2 influenza pandemics.^6^

H6-subtype IAVs have been detected in various migratory waterfowl and domestic poultry in Eurasia and North America.^7^ Most H6 viruses introduced from waterfowl into domestic poultry have gained only limited spread. However, during 2000–2005, subtype H6N2 IAVs caused illness outbreaks among domestic poultry in CA, USA.^8, 9, 10^ In addition, H6 IAVs have been shown to replicate well in mice without pre-adaptation, indicating that these viruses could cause cross-species infection in mammals.^11, 12^ Laboratory experiments showed that humans can be infected with H6 IAVs through experimental inoculation.^13^ Furthermore, findings from serologic surveillance suggested that veterinarians exposed to H6 IAV-infected domestic birds can become infected with the virus,^14^ and in 2013, an avian-origin H6N1 IAV was reported to cause human infection, but there has been no evidence of subsequent human-to-human transmission.^15^

Since 2002, H6 IAVs have been one of the predominant IAV subtypes circulating in live bird markets in southern China,^16, 17, 18^ and some of these H6 viruses recognized human receptors.^19^ In 2010, after an avian-origin H6N6 swine influenza A virus (SIV) was isolated from sick pigs in southern China, it was found that the virus had been transmitted to and was circulating among the swine population; seroprevalence rates ranged from 1.8% to 3.4%.^20, 21^ The hemagglutinin (HA) protein of the currently circulating H6N6 SIV has amino acids 222V and 228S, compared with amino acids 222A and 228G in its potential AIV precursors. In other IAV subtypes, HA amino acids 222V and 228S have been reported to affect virus replication in mammals.^7, 22^

The virus that caused the 2009 H1N1 pandemic, influenza A(H1N1)pdm09, was a swine-origin IAV.^23^ After its discovery in humans, this virus quickly moved to swine and other animal populations worldwide.^24, 25, 26, 27, 28^ During the past few years in southern China, A(H1N1)pdm09 virus has become one of the predominant viruses among domestic swine.^29, 30, 31^ Frequent reassortments between influenza A(H1N1)pdm09 and other endemic SIVs have been observed.^32, 33^

The aims of our study were to understand the impacts of two acquired mutations in HA of H6N6 virus on its receptor-binding properties and to assess the transmission potential of the H6N6 virus. We also aimed to assess the potential risks posed by reassortants of H6N6 virus with A(H1N1)pdm09 virus because such reassortants would be expected to emerge if both viruses continue to circulate in swine.

## MATERIALS AND METHODS

### Virus and RNA extraction

In 2010, an avian-like H6N6 SIV, A/swine/Guangdong/k6/2010(H6N6), was isolated from swine in southern China.^20^ To study the virus, we used an RNeasy Mini Kit (Qiagen, Germantown, MD, USA) to extract RNA from the isolate; extraction was performed in a Biosafety Level 3 (BSL-3) laboratory.

### Cells

Madin-Darby canine kidney (MDCK) cells and human embryonic kidney 293 T cells (both from American Type Culture Collection, Manassas, VA, USA) were used for virus propagation; the cells were maintained in Dulbecco's Modified Eagle Medium (DMEM; Gibco/BRL, Grand island, NY, USA). A549 cells (American Type Culture Collection) used in assays were maintained in Advanced DMEM/F-12 (Gibco/BRL). The medium for each of the three cell lines was supplemented with 10% fetal bovine serum (Atlanta Biologicals, Lawrenceville, GA, USA), penicillin–streptomycin, and amphotericin B (Gibco/BRL), and the cells were held at 37 °C in 5% CO_2_.

### Molecular cloning, mutagenesis and reverse genetics

The full-length cDNA for eight genes of A/swine/Guangdong/k6/2010(H6N6) virus were amplified by using the SuperScript One-Step RT-PCR system (Invitrogen, Carlsbad, CA, USA) and then cloned into a pHW2000 vector.^34^ The site-directed mutagenesis on residues 222 and 228 of HA was performed using a QuikChange II Site-Directed Mutagenesis Kit (Stratagene, La Jolla, CA, USA). Primers are available upon request.

Eleven recombinant viruses were generated (Table 1) by reverse genetics as previously described.^35^ The recombinant viruses were confirmed by Sanger sequencing at the Life Sciences Core Laboratories Center at Cornell University (Ithaca, NY, USA).

### Hemagglutination and hemagglutination inhibition assays

Hemagglutination and hemagglutination inhibition (HI) assays were carried out by using 0.5% turkey erythrocytes as previously described.^35^

### Glycans

The biotinlyated α2,3- and α2,6-sialic acid receptors (3′SLN and 6′SLN, respectively) were purchased from GlycoTech (Gaithersburg, MD, USA). *N*-linked glycans, Manα1,6-(Neu5Acα2,3-Galβ1,4-GlcNAcβ1,2-Manα1,3-)Manβ1,4-GlcNAcβ1,4-GlcNAc (N32) and Neu5Acα2,3-Galβ1,4-GlcNAcβ1, 2-Manα1,6-(Manα1,3-)Manβ1,4-GlcNAcβ1,4-GlcNAc (N52) were synthesized to represent as *N*-acetylneuraminic acid-α2,3-linked galactose (Neu5Acα2,3-Gal) and Manα1,6-(Neu5Acα2,6-Galβ1,4-GlcNAcβ1,2-Manα1,3-)Manβ1,4-GlcNAcβ1,4-GlcNAc (N33) and Neu5Acα2,6-Galβ1,4-GlcNAcβ1,2-Manα1,6-(Manα1,3-)Manβ1,4-GlcNAcβ1,4-GlcNAc (N53) were synthesized to represent as *N*-acetylneuraminic acid-α2,6-linked galactose (Neu5Acα-2,6-Gal). The *N*-linked glycans were first labeled by 2-amino-*N*-(2-amino-ethyl)-benzamide (AEAB) as described previously^36^ and then biotinlyated by using EZ-Link NHS-LC-LC-Biotin (Thermo Fisher Scientific, Waltham, MA, USA) according to the manufacturer's instruction. Glycan quantities were measured by high-performance liquid chromatography (Shimadzu, Columbia, MD, USA).

### Virus glycan receptor-binding assay

The glycan stock solution (1 mg/mL) was prepared in 50% glycerol in 1 × phosphate-buffered saline (PBS) (v/v), according to the manufacturer's instructions. The protein concentration in viruses was determined using a Pierce BCA Protein Assay Kit (Thermo Fisher Scientific) according to the manufacturer's instructions. For the binding analysis, we further diluted the sialic acid receptors (*N*-linked glycans, 3′SLN and 6′SLN) and the viruses in a PBS solution (pH=7.4) containing 0.01% bovine serum albumin and 0.002% Tween-20 (1 × Kinetics Buffer 10 × ; FortéBIO Inc., Menlo Park, CA, USA) with 10 μM neuraminidase inhibitor (zanamivir hydrate; Moravek Inc., Brea, CA, USA) and 10 μM oseltamivir phosphate (American Radiolabeled Chemicals Inc., St Louis, MO, USA). The binding assay was performed by using a FortéBIO Octet K2 interferometer equipped with streptavidin biosensor tips (FortéBIO Inc.). In summary, the biotinylated receptors were first coated onto the biosensor tips for 300 s, after which the tips were dipped into a 1 mg/mL protein concentration of virus for 1200 s (association step) and then into the kinetics buffer with neuraminidase inhibitors for 1000 s (dissociation step). The entire measurement cycle was maintained at 30 °C with orbital shaking at 1000 × rpm.

### Growth kinetics and plaque assays

To determine the growth kinetics, we inoculated the MDCK and A549 cells with rgH6N6-222V/228S, rgH6N6 × pdm09-222V/228S, rgH6N6-222A/228G, rgH6N6 × pdm09-222A/228G, rgH3N2 × pdm09 (Table 1), wild-type A/California/04/2009(H1N1), or wild-type A/swine/Ohio/11SW226/2011(H3N2) at a multiplicity of infection of 0.001 (for MDCK cells) or 0.1 (for A549 cells). After the cells were incubated at 37 °C for 1 h, the inocula were removed. Cells were then washed twice with PBS and incubated for 96 h at 37 °C in 5% CO_2_ with 1.5 μg/mL Opti-MEM I (Life Technologies, Carlsbad, CA, USA) or Opti-MEM I containing TPCK-treated trypsin. At 12, 24, 48 and 72 h after inoculation, supernatant (200 μL) was collected from the cells and titrated, by 50% tissue culture infectious dose (TCID_50_), in MDCK cells.

Plaque assays were performed on MDCK cells in six-well tissue culture plates. Serial dilutions were prepared from the virus stock, and 800 μL of each dilution was incubated in MDCK cells at 37 °C with 5% CO_2_ for 1 h. The inocula were then aspirated, and the cells were overlaid with 2 mL of 1% agarose containing TPCK-treated trypsin (1.5 μg/mL). Cultures were incubated for 3 days at 37 °C and then fixed with methanol and stained with 1% crystal violet to reveal plaques.

### Glycan microarray and data analyses

The viruses were purified using 25% sucrose as previously described.^37^ The virus labeling, glycan microarray hybridization and data analyses were performed as previously described.^38^

### Animal experiments

To test the transmissibility of the two testing viruses (rgH6N6-222V/228S and rgH6N6 × pdm09-222V/228S), we designed six experiment groups for each virus: three aerosol transmission and three direct contact transmission groups. Two 4-month-old female ferrets (Triple F Farms) were included in each of the 12 groups: one as a virus-inoculated ferret to be inoculated intranasally with a testing virus (10^6^ TCID_50_ viral load in a 1-mL volume) and the other as an exposure ferret to be exposed to the virus through indirect (that is, aerosol) or direct contact with the virus-inoculated ferret. Before the experiments were conducted, all 24 ferrets tested negative for antibodies to rgH6N6-222V/228S (wild-type-like), A/California/04/2009(H1N1), A/Perth/16/2009(H3N2), A/Victoria/361/2011(H3N2) and A/Minnesota/307875/2012(H3N2) influenza viruses. In the aerosol transmission groups, the virus-inoculated and exposure ferrets were housed in the same cage on different sides of a 1-cm-thick, double-layered, steel partition with 5-mm perforations (Allentown Inc., Allentown, NJ, USA). The airflow in the cage went from the exposure ferret to the virus-inoculated ferret. In the direct-contact transmission groups, the virus-inoculated and exposure ferrets were housed together in the same cage without a partition. In all cages, the exposure ferret was put into the cage 1 day after the virus-inoculated ferret was inoculated with a testing virus.

Nasal wash fluids were collected from virus-inoculated ferrets at one and two days post inoculation (DPI) and from exposure ferrets at 1 DPI; thereafter, nasal wash fluids were collected every other day until 10 DPI for both groups of ferrets. Body temperature and weight were measured before nasal wash fluids were collected.

Serum samples were collected from all ferrets at 14 DPI, immediately before they were killed. Virus titers in nasal wash fluids were determined by TCID_50_ in MDCK cells and confirmed by 50% egg infectious dose (EID_50_) in 9-day-old embryonated chicken eggs.

To test the replication efficiency of testing viruses in the ferret respiratory track, we killed two of the three virus-inoculated ferrets in each contact transmission group at 3 DPI. The turbinates, trachea, bronchi and lungs were collected, and virus titers were determined by TCID_50_ in MDCK cells.

### Biosafety and animal handling

All laboratory and animal experiments were conducted under BSL-2 conditions, with investigators wearing appropriate protective equipment, and in compliance with protocols approved by the Institutional Animal Care and Use Committee (IACUC) of Mississippi State University.

### Phylogenetic analyses

We conducted multiple sequence alignments by using the MUSCLE software package.^39^ We used GARLI version 0.96^40^ and maximum likelihood criteria to perform phylogenetic analyses, and we conducted bootstrap resampling analyses with 1000 runs by using PAUP* 4.0 Beta^41^ with a neighbor-joining method as described elsewhere.^42^

## RESULTS

### H6N6 SIV differs genetically from the H6N1 AIV that infected humans

**Figure 1:**
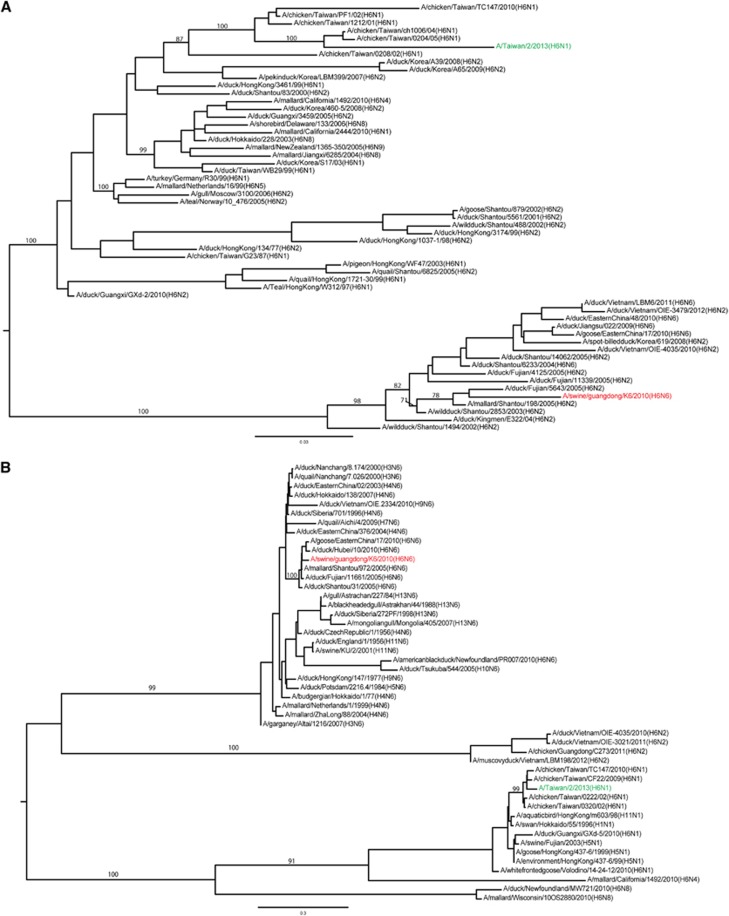

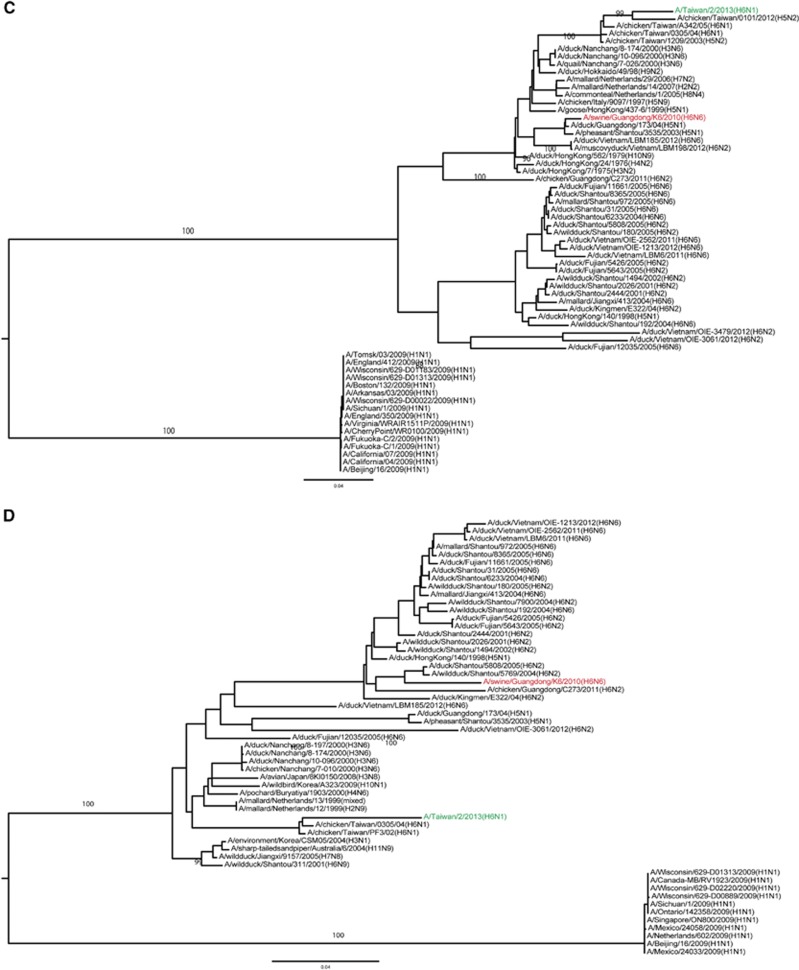

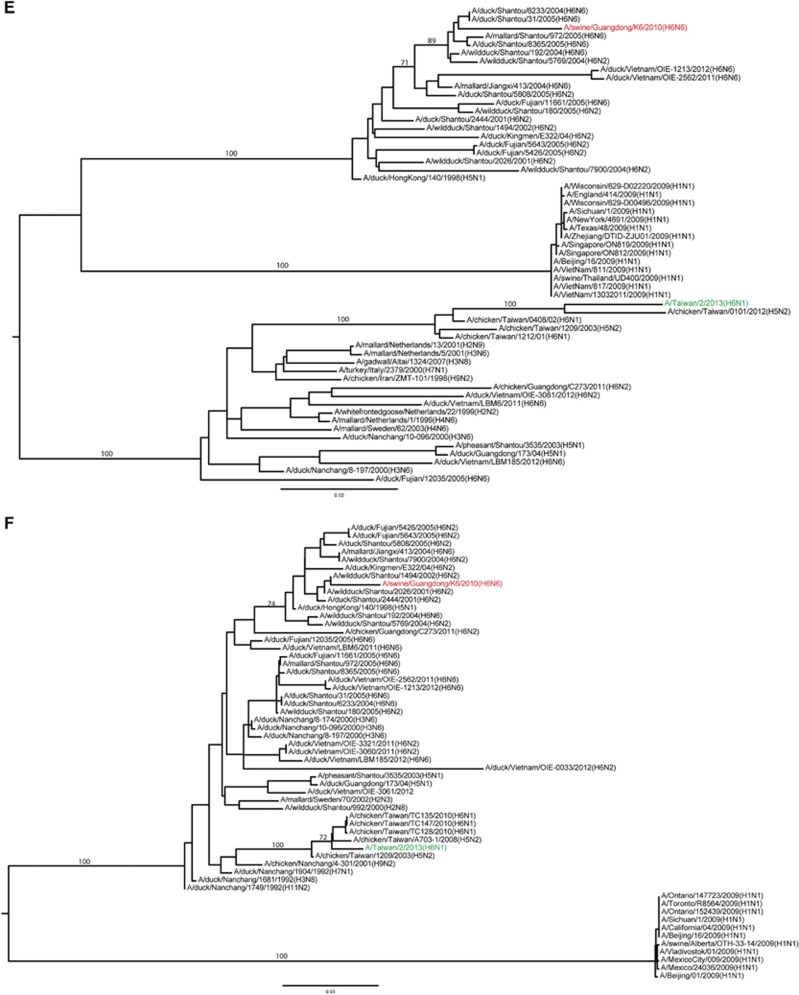

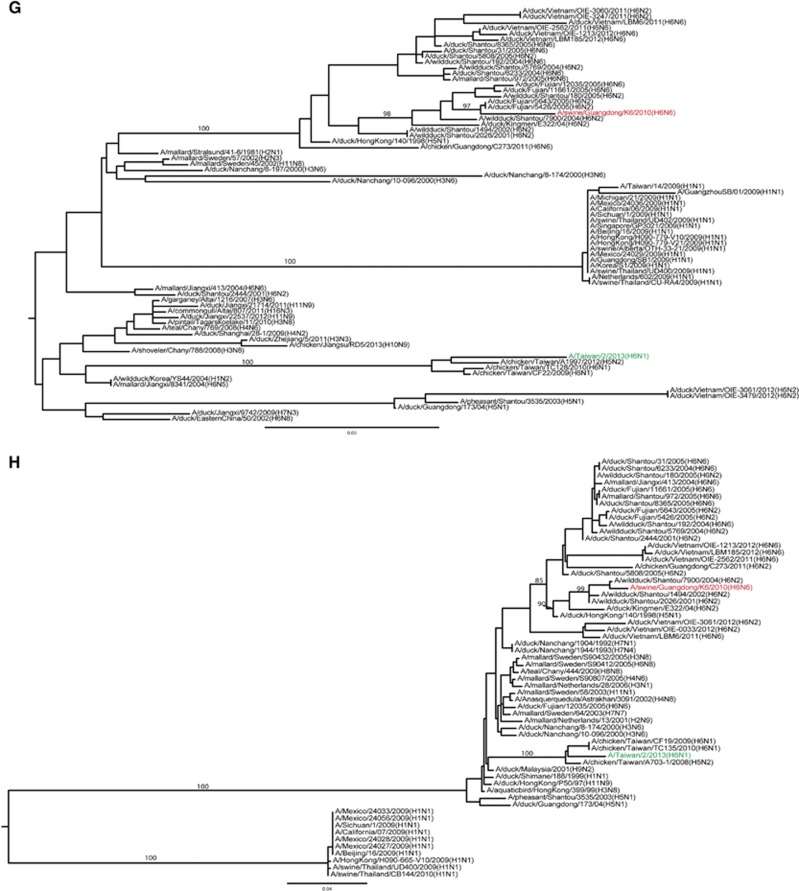
Phylogenetic analyses of subtype H6N6 swine influenza virus A/swine/Guangdong/k6/2010(H6N6). (**A**) HA gene. (**B**) NA gene. (**C**) PB2 gene. (**D**) PB1 gene. (**E**) PA gene. (**F**) NP gene. (**G**) MP gene. (**H**) NS gene. The phylogenetic trees were constructed by using maximum-likelihood implemented in GARLI version 0.96;^40^ bootstrap values were generated, with 1000 replications, by using neighbor-joining methods implemented in PAUP* 4.0 Beta.^41^ Scale bars represent nucleotide substitutions per site. The virus marked in red indicates the H6N6 strain isolated from swine; the virus marked in green indicates the subtype H6N1 avian influenza strain isolated from a human in Taiwan.

Phylogenetic analyses showed that the HA genes from H6N6 SIVs and H6 AIVs, including the strain that caused human infection, belong to different sublineages within a Eurasian lineage (Figure 1A). The PB2, NP and NS genes of H6N6 and H6N1 viruses belong to the same genetic lineages, but the PB1, PA and MP genes belong to different lineages (Figures 1B–1H). None of these genes was genetically close to those of influenza A(H1N1)pdm09 virus or other circulating H3N2 and H1N1 SIVs in southern China (Figures 1B–1H). The HA protein in the H6N6 SIV is 73.2% identical to that of the H6N1 AIV that was isolated from a human in southern China. The HA in the H6N6 SIV has amino acids 222V and 228S, whereas the HA in the human H6N1 AIV has amino acids 222A and 228S; the corresponding progenitors of these SIVs (that is, subtype H6 AIVs) have amino acids 222A and 228G.

### Substitution G228S but not A222V increases binding affinity of H6N6 viruses to guinea pig and horse erythrocytes

To investigate whether substitutions A222V and G228S (avian to swine) affect virus-binding affinity to erythrocytes, we conducted hemagglutination assays with eight reassortant viruses generated by reverse genetics: rgH6N6 × PR8-222V/228S, rgH6N6 × PR8-222A/228S, rgH6N6 × PR8-222V/228G, rgH6N6 × PR8-222A/228G, rgH6N6 × pdm09-222V/228S, rgH6N6 × pdm09-222A/228S, rgH6N6 × pdm09-222V/228G, and rgH6N6 × pdm09-222A/228G (Table 1). Erythrocytes from chicken, turkey, guinea pigs and horses were used. All eight viruses were normalized to a hemagglutination titer of 32 by using turkey erythrocytes (Table 2). The rgH6N6 × PR8-222V/228S, rgH6N6 × pdm09-222V/228S, rgH6N6 × PR8-222A/228S and rgH6N6 × pdm09-222A/228S viruses had hemagglutination titers of 16 or 32 against chicken, guinea pig and horse erythrocytes. However, the rgH6N6 × PR8-222V/228G, rgH6N6 × pdm09-222V/228G, rgH6N6 × PR8-222A/228G and rgH6N6 × pdm09-222A/228G viruses had a hemagglutination titer of ≤2 to guinea pig and horse erythrocytes and a hemagglutination titer of ≥64 to chicken erythrocytes. Thus, substitution G228S increased the binding affinity of H6N6 virus to guinea pig and horse erythrocytes.

**Figure 2:**
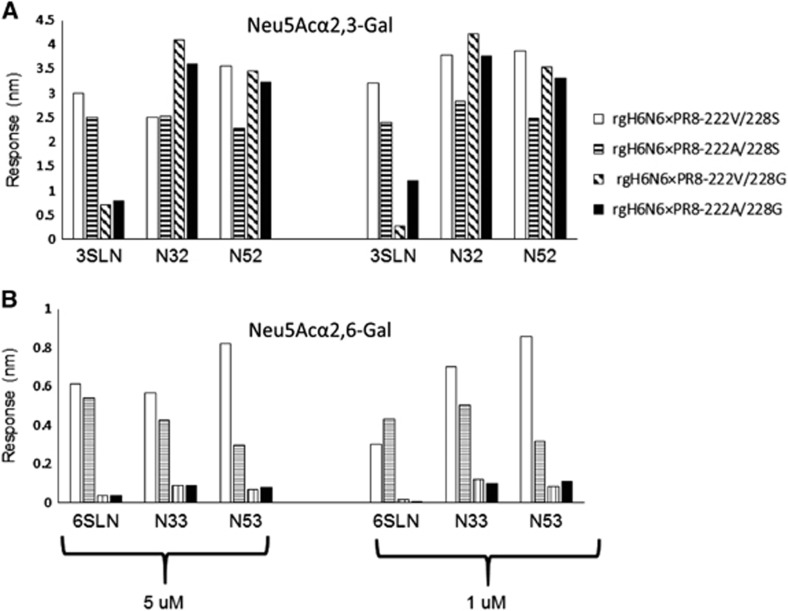
Virus-binding affinities of recombinant influenza A(H6N6) viruses to glycans. Binding affinities of viruses to specific biotinylated Neu5Acα2,3-Gal glycans (**A**) and to specific biotinylated Neu5Acα2,6-Gal glycans (**B**). Four viruses were used: rgH6N6 × PR8-222V/228S, rgH6N6 × PR8-222A/228S, rgH6N6 × PR8-222V/228G and rgH6N6 × PR8-222A/228G. Six biotinlyated glycans were used, including two analogs (3′SLN and 6′SLN) and four synthetic *N*-linked glycans (N32, N33, N52 and N53). 3′SLN, N32 and N52 were used to represent as Neu5Acα2,3-Gal; 6′SLN, N33 and N53 were used to represent as Neu5Acα2,6-Gal.

To further determine the effects of mutations A222V and G228S (avian to swine) on the receptor-binding properties of H6N6 virus, we also performed binding assays using a FortéBIO system with a set of Neu5Acα2,3-Gal and Neu5Acα2,6-Gal glycans: two glycan analogs (3′SLN and 6′SLN) and four synthetic *N*-linked glycans (N32, N33, N52 and N53). Results showed that the rgH6N6-222V/228S (wild-type-like) virus can bind to Neu5Acα2,3-Gal (3′SLN, N32 and N52) and Neu5Acα2,6-Gal (6′SLN, N33 and N53), but the affinities to Neu5Acα2,3-Gal (3′SLN, N32 and N52) were at least two-fold higher than those to Neu5Acα2,6-Gal (6′SLN, N33 and N53; Figure 2). Binding affinities for the avian-like mutants rgH6N6-222V/228G and rgH6N6-222A/228G to the Neu5Acα2,6-Gal glycans (6′SLN, N33 and N53) were reduced by more than 80% compared with those for rgH6N6-222V/228S, but binding affinities to two synthetic Neu5Acα2,3-Gal glycans (3′SLN, N32 and N52) were not reduced to the same extent. The binding affinities of avian-like mutant rgH6N6-222A/228S to Neu5Acα2,3-Gal (3′SLN, N32 and N52) and Neu5Acα2,6-Gal (6′SLN, N33 and N53) were reduced by different extents (Figure 2). In summary, mutation G228S (avian to swine) increased the binding affinity of H6N6 IAV to the testing Neu5Acα2,6-Gal glycans (6′SLN, N33 and N53) and Neu5Acα2,3-Gal (3′SLN) but not to Neu5Acα2,3-Gal (N32 and N52). Compared with mutation G228S, mutation A222V (avian to swine) had much less effect on binding affinities to Neu5Acα2,3-Gal and Neu5Acα2,6-Gal glycans.

### H6N6 virus binds to α2,3- and α2,6-linked sialic acid receptors, and mutations A222V and G228S affect virus receptor affinity

**Figure 3:**
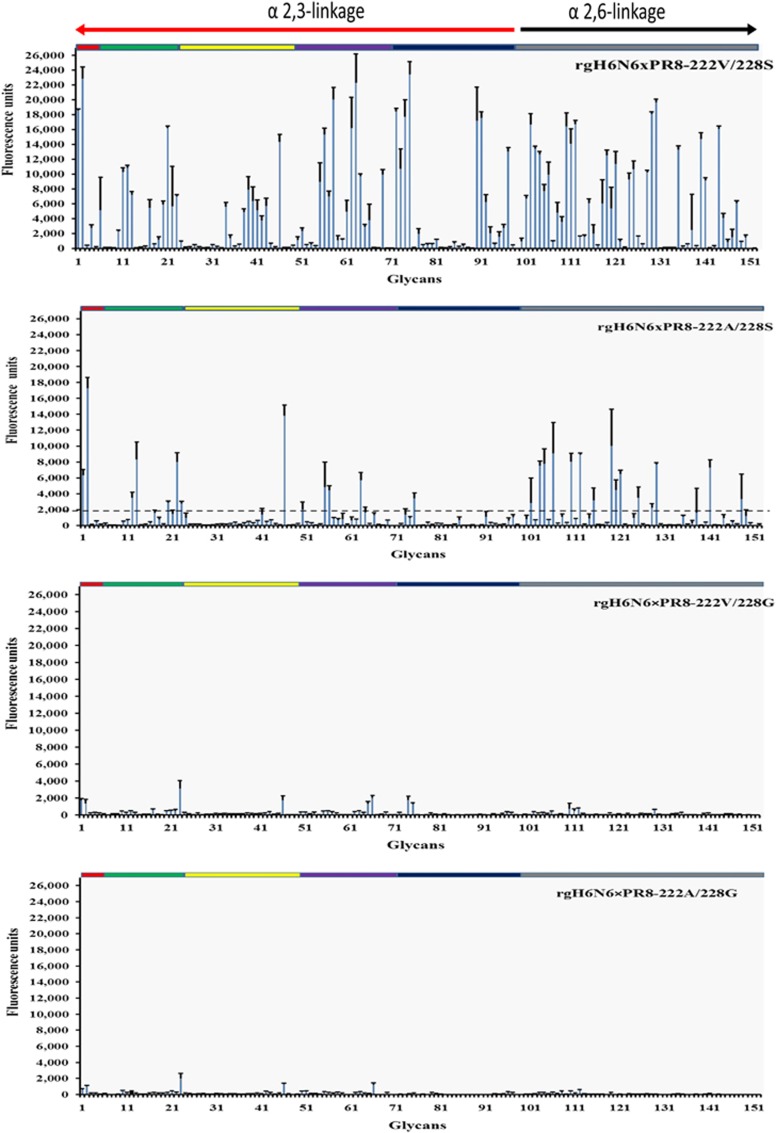
The binding profile of recombinant influenza A(H6N6) viruses to sialic acids on the glycan array. Colors indicate different categories of glycans on the array: red, α2,3-sulfated sialosides; green, α2,3- di-, tri- and qua-sialosides; yellow, α2,3-linear sialosides; purple, α2,3-fucosylated sialosides; blue, α2,3-internal sialosides; black, α2,3- and α2,6-sialosides; and gray, different categories of α2,6-sialosides are highlighted in the same order as listed for the α2,3-sialosides. The dashed line indicates minimum relative fluorescence units of 2000. Black bars represent error bars. The glycan sequences are detailed in Supplementary Table S1.

The glycan array, which contained a total of 152 α2,3-linked and α2,6-linked glycans, was used to determine the receptor-binding profile of H6N6 SIV and the effect of mutations V222A and S228G on the glycan-binding profile of H6N6 IAV. Results showed that rgH6N6 × PR8-222V/228G and rgH6N6 × PR8-222A/228G had binding affinities below the detection threshold for the majority of the α2,3-linked and α2,6-linked glycans on the glycan array (Figure 3). However, rgH6N6 × PR8-222V/228S showed high affinity for binding to 98 of the α2,3-linked and 54 of the α2,6-linked glycans; binding affinities were above the detection threshold of 2000 mean relative fluorescence units. Furthermore, the binding affinities to α2,3-linked and α2,6-linked glycans for rgH6N6 × PR8-222A/228S were weaker than those for rgH6N6 × PR8-222V/228S but higher than those for rgH6N6 × PR8-222V/228G and rgH6N6 × PR8-222A/228G. These results suggest double mutations A222V and G228S increased the binding affinities of H6N6 virus to the sialic acid glycans used in the glycan arrays.

### Transmission of H6N6 wild-type SIV and rgH6N6 virus possible between ferrets

**Figure 4:**
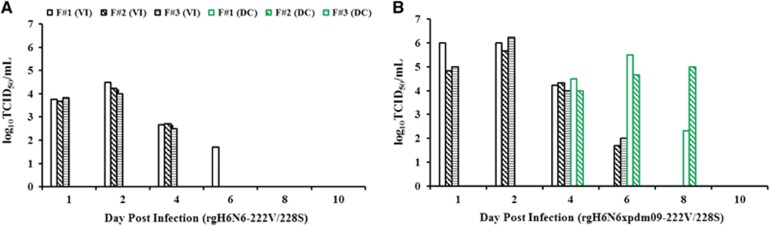
Mean titers of influenza viruses recovered from nasal wash fluids of virus inoculated and contact ferrets in direct-contact transmission experiments. Inoculated ferrets were nasally inoculated with 10^6^ TCID_50_ of each virus shown: the wild-type-like rgH6N6-222V/228S (**A**) and rgH6N6xpdm09-222V/228S (**B**), and 1 day later each was housed in the same cage with a contact ferret. Virus titers were measured in nasal wash fluids (collected on indicated days) by using endpoint titration in Madin-Darby canine kidney cells; ending titers were expressed as mean log_10_TCID_50_/mL±standard deviation. The limit of virus detection was 10^0.699^ TCID_50_/mL. the ferret #1, F#1; the ferret #2, F#2; the ferret #3, F#3; virus inoculated, VI; direct contact, DC.

We used a ferret model to determine the transmissibility of rgH6N6-222V/228S virus by direct and indirect (aerosol) contact. In the direct-contact transmission experiment, the rgH6N6-222V/228S virus-inoculated ferrets did not show obvious clinical signs of illness. At 1 DPI, nasal wash fluids from these ferrets had virus titers ranging from 10^3.67^ to 10^3.83^ TCID_50_/mL, and at 2 DPI, titers peaked at 10^4.5^ TCID_50_/mL; viral shedding continued until 5 DPI in these ferrets (Figure 4). HI assay results showed that serum collected from these virus-inoculated ferrets at 14 DPI had virus titers ranging from 1:320 to 1:1280, indicating all ferrets seroconverted (Table 3). Ferrets exposed to the virus-inoculated ferrets through direct contact had no detectable viral shedding when MDCK cells were used for detection; however, one of the three direct-contact ferrets showed seroconversion (Table 3).

**Figure 5:**
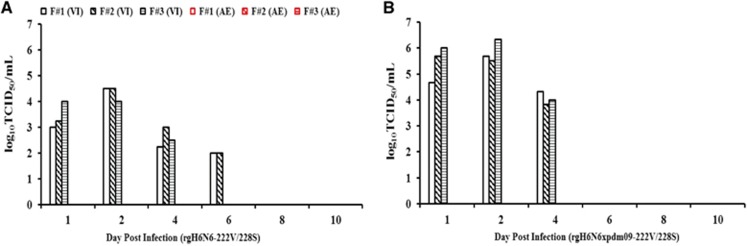
Mean titers of influenza viruses recovered from nasal wash fluids of virus-inoculated and contact ferrets in aerosol transmission experiments. Inoculated ferrets were inoculated with 10^6^ 50% tissue culture infectious doses (TCID_50_) of each virus shown: the wild-type-like rgH6N6-222V/228S (**A**) and rgH6N6xpdm-222V/228S (**B**), and 1 day later, a contact ferret was housed in the same cage as an inoculated ferret but on a different side of a 1-cm-thick, double-layered, steel partition with 5-mm perforations (Allentown Inc.). Virus titers were measured in nasal wash fluids (collected on indicated days) by using endpoint titration in Madin-Darby canine kidney cells; ending titers were expressed as mean log_10_TCID_50_/mL±standard deviation. The limit of virus detection was 10^0.699^ TCID_50_/mL. aerosol exposure, AE; the ferret #1, F#1; the ferret #2, F#2; the ferret #3, F#3; virus inoculated, VI.

As in the direct-contact transmission experiment, rgH6N6-222V/228S virus-inoculated ferrets in the aerosol transmission study did not exhibit clinical signs of illness. At 1 DPI, nasal wash fluids from these virus-inoculated ferrets had median rgH6N6 virus titers ranging from 10^3.00^ to 10^4.00^ TCID_50_/mL, and virus titers peaked at 2 DPI at 10^4.50^ TCID_50_/mL; these virus-inoculated ferrets continued to shed viruses until 6 DPI (Figure 5). HI assay results showed that serum collected from these virus-inoculated ferrets at 14 DPI had virus titers ranging from 1:320 to 1:1280, indicating all ferrets had seroconverted (Table 3). None of the three exposure ferrets in the aerosol transmission study had detectable viral shedding when MDCK cells were used as the detection method, and HI assay results showed that none of the ferrets had seroconverted before being killed at 14 DPE (Table 3). However, when we used embryonated chicken eggs as the detection method, the nasal wash fluids collected from one of the three ferrets at 6 and 8 DPE had an EID_50_ of 10 (Table 4).

To validate the aerosol transmissibility of this H6N6 virus, we repeated the experiment with the wild-type H6N6 isolate at an animal BSL-3 facility. The results from this independent experiment showed that the wild-type H6N6 isolate caused seroconversion in only one of the two direct-contact exposure ferrets and one of the two aerosol-exposure ferrets (data not shown), supporting that H6N6 virus has limited transmissibility between ferrets through direct contact or through inhalation of infectious aerosolized droplets.

### Internal genes of influenza A(H1N1)pdm09 virus did not facilitate transmission of H6N6 virus among ferrets

To assess the risks posed by a potential reassortant rgH6N6 × pdm09 strain, which could result from co-circulating H6N6 SIV and influenza A(H1N1)pdm09 virus, we determined transmissibility of the reassortant virus by direct contact and aerosol contact in ferrets. The three rgH6N6 × pdm09-222S/228V virus-inoculated ferrets had weight loss and slightly elevated body temperatures at 3 DPI, but the ferrets showed clinical recovery from 4 DPI onward. Nasal wash fluids collected from the virus-inoculated ferrets at 1 DPI had virus titers of 10^4.67^–10^6.00^ TCID_50_/mL; at 2 DPI, virus titers peaked at 10^6.33^ TCID_50_/mL and continued to shed until 6 DPI (Figure 5). All three virus-inoculated ferrets seroconverted, with HI titers ranging from 1:640 to 1:1280, at 14 DPI (Table 3).

In the direct-contact transmission experiment, two of the three exposure ferrets had no overt signs of illness. However, these ferrets had detectable virus loads (range, 10^4.00^–10^4.50^ TCID_50_/mL in nasal wash fluids at 4 DPE (Figure 4), and viral shedding was sustained for at least 5 days. All three direct-contact exposure ferrets seroconverted, with HI titers of 1:640, at 14 DPE (Table 3).

In the aerosol transmission experiment, the exposure ferrets had no detectable viral shedding when MDCK cells were used as the detection method. However, the nasal wash fluids collected from one of the three exposure ferrets at 2 DPE had a virus titer of 10 EID_50_/mL when embryonated chicken eggs were used for detection (Figure 5; Table 4), but none of these exposure ferrets seroconverted by 14 DPE (Table 3).

### Internal genes of influenza A(H1N1)pdm09 virus increased replication efficiency of H6N6 virus in ferret lower respiratory tract

**Figure 6:**
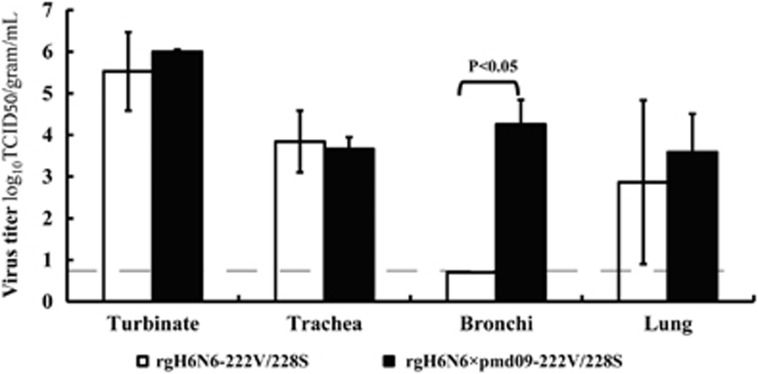
Mean titers of influenza viruses recovered from respiratory tract tissues of ferrets after nasal inoculation of virus. Ferrets were inoculated with 10^6^ 50% tissue culture infectious doses (TCID_50_) of each virus shown: the wild-type-like rgH6N6-222V/228S and rgH6N6xpdm09-222V/228S. Two ferrets were killed three days after inoculation, and virus titers in nasal turbinate, trachea, bronchus and lung of each ferret were determined by using end-point titration in Madin-Darby canine kidney cells. The titers were quantified as the mean titer from at least three sections of each tissue. The limit of virus detection was 10^0.699^ TCID_50_/mL. The dashed line indicates the lower limit of detection. Black bars represent error bars.

To evaluate the effects of the internal genes on the pathogenesis of H6N6 virus, we inoculated ferrets with rgH6N6-222S/228G and rgH6N6 × pdm09-222S/228G viruses and compared the replication efficiencies of the viruses in ferret respiratory track tissues. Results showed rgH6N6-222S/228G replicated without prior adaptation in ferret nasal turbinate, trachea and lung but not in bronchi (Figure 6). At 3 DPI, the turbinate, trachea and lung tissues of the ferrets inoculated with rgH6N6-222S/228G (wild-type) virus had virus titers of 10^5.53^, 10^3.84^ and 10^2.87^ TCID_50_/g, respectively. In the ferrets inoculated with rgH6N6 × pdm09-222S/228G virus, the turbinate, trachea, bronchi and lung tissues at 3 DPI had virus titers of 10^6.00^, 10^3.67^, 10^4.26^ and 10^3.59^ TCID_50_/g, respectively. Thus, the virus titers in respiratory tract tissues, especially lower respiratory tract tissues, such as bronchi and lung, from the rgH6N6 × pdm09-222S/228G virus-inoculated ferrets was five-fold higher than those from rgH6N6-222S/228G virus-inoculated ferrets.

## DISCUSSION

Together, the demonstrated promiscuous nature of H6 AIVs, their prevalence in southern China, and the case of H6N1 virus infection in a human in southern China ^15^ raise concerns that the H6N6 SIV emerging in that area's swine population could become or contribute to an enzootic influenza strain causing human infections. Such a transition could occur through the commonplace evolutionary events in influenza viruses, such as acquisition of adapted mutations or entire gene segments from currently co-circulating SIVs. Phylogenetic analyses showed that the H6N6 virus is genetically different from the H6N1 virus that infected a human in Taiwan. These findings suggest that the H6 IAVs in southern China are genetically diverse,^16, 17, 18^ and active evolutionary events that are still ongoing among the H6 AIVs have led to the emergence of an H6N1 virus in a human^15, 43, 44^ and to H6N6 viruses in swine.^20^ Virus mutation and reassortment rates have been key measures in virologic risk assessments of influenza.^45^ The presence of genetically diverse H6 IAVs and active evolutionary events increases the possibility for a virus of this subtype to develop pandemic potential and present a risk to public health.

Compared with its progenitor AIV, H6N6 SIV has mutations 222V and 228S in the 220-loop of its HA. Mutations in this 220-loop (for example, at residues 222, 225, 226 and 228) have been shown to affect receptor-binding specificity. For example, mutations Q226L and G228S enabled H3 viruses to bind sialic acid α2,3-Gal and sialic acid α2,6-Gal receptors.^46^ The D222G substitution (corresponding to residue 225 in H3 viruses) enabled influenza A(H1N1)pdm09 viruses to acquire dual receptor specificity for complex α2,3-linked and α2,6-linked sialic acids; the substitution also increased the virulence of this virus.^47^ We recently showed that the W222L substitution in HA could facilitate infection of H3N2 IAV in dogs, possibly by increasing the binding affinity of the virus to canine-specific receptors with Neu5Acα2,3-Galβ1,4-(Fucα-) or Neu5Acα2,3-Galβ1,3-(Fucα-)-like structures.^38^ In addition, mutation W222R in HA can increase influenza virus infectivity in mice^48, 49^ by introducing a hydrogen bond between the virus HA and the host glycan receptor.^50^

Previous studies showed that turkey and chicken erythrocytes express α2,3- and α2,6-linked sialic acids that horse erythrocytes almost exclusively express α2,3-linked sialic acids,^51^ and that guinea pig erythrocytes disproportionately express α2,3- and α2,6-linked sialic acids.^52^ The receptor-binding profiles we obtained in this study with turkey, chicken, guinea pig, dog and horse erythrocytes suggest that mutations V222A and G228S, which are associated with a change in affinity from avian to swine, especially mutation G228S, changed receptor-binding specificity. This mutation G228S led to a minimum 16-fold increase of receptor-binding affinities for guinea pig and horse erythrocytes and a minimum two-fold decrease in binding affinities to chicken erythrocytes (Table 2). Thus, mutation G228S could affect virus binding to both α2,3-linked and α2,6-linked sialic acids, but the effects on α2,6-linked sialic acids were significantly higher than that on α2,3-linked sialic acids. These results were confirmed by the virus glycan-binding assay results, which showed that wild-type H6N6 virus can bind to α2,3- and α2,6-linked sialic acids and that substitution G228S (avian to swine) significantly increased the binding affinities of H6N6 IAV to three α2,6-linked sialic acids that we tested (Figure 2). In addition, glycan microarray data analysis confirmed that mutations A222V and G228S (avian to swine) can increase HA-binding affinities to glycans, including α2,6-linked sialic acid (Figure 3). The results from virus glycan binding were consistent with those using recombinant HA proteins of H6N1 human influenza virus, which has amino acid 228S.^53^ In summary, these findings indicated that mutations A222V and especially G228S, could facilitate the binding ability of H6N6 virus to α2,6-linked sialic acids. The conservation of S228 between the H6N1 human isolate and H6N6 SIV suggests that the G228S mutation likely facilitated transmission of H6 IAV to swine or humans.

In H6N6 SIV-infected and rgH6N6 virus-infected ferrets, virus was shed at high titers until 6 DPI, similar to viral shedding by other H6 viruses.^11, 12^ Previous studies in ferrets showed that H6N2 AIVs replication was more efficiently in lungs than nasal turbinates at 5 DPI.^11^ However, our findings show that the minimum H6N6 SIV load in nasal turbinates was 10-fold higher than that in lungs at 3 DPI (Figure 6). In our experiments, it is likely that H6N6 SIV replicated better in the upper than lower respiratory tract of ferrets; such a situation would lead to constant viral shedding and to transmissibility of this virus among ferrets. Additional experiments are needed to understand whether mutations A222V and G228S (avian to swine) alter viral tissue tropisms in ferrets.

A number of studies have shown that AIVs, such as highly pathogenic H5N1 and low pathogenicity H9N2 viruses, have increased aerosol transmissibility after acquiring the internal genes of influenza A(H1N1)pdm09 virus.^54, 55, 56^ However, the results of our transmission studies showed that the internal genes of A(H1N1)pdm09 virus did not increase transmissibility of H6N6 SIVs through aerosol or direct contact. Nonetheless, it is possible that additional mutations acquired through adaptation or genetic reassortments between H6N6 SIV and influenza A(H1N1)pdm09 virus would increase the transmissibility of H6N6 SIV.

In the pathogenesis studies, virus replicated in lungs of rgH6N6 virus–infected ferrets, but the ferrets exhibited no obvious signs of illness. Virus titers in lungs of rgH6N6 × pdm09 virus-infected ferrets were ≥5-fold higher than those in rgH6N6 virus-infected ferrets. In addition, ferrets infected with rgH6N6 × pdm09 virus showed slightly elevated temperatures and weight loss at 1 and 2 DPI.

We showed that the method used for detecting virus loads in nasal wash fluids or tissues affects the accuracy of the results. For example, embryonated chicken eggs were more sensitive than MDCK cells in detecting H6N6 SIVs in the virus transmission group: viruses in aerosol-exposed ferrets were detectable in embryonated chicken eggs but not in MDCK cells (Table 4).

In summary, our findings suggest that subtype H6N6 virus can bind to α2,6-linked sialic acids, indicating H6N6 virus as a virus with zoonotic potential. Although H6N6 SIV has limited transmissibility between ferrets and probably cannot yet be transmitted between ferrets through infectious aerosolized droplets, the virus could evolve into a more transmissible H6 virus through additional adaptation and reassortment. Thus, evolution of this H6N6 virus and other H6 AIVs should be closely monitored.

## Figures and Tables

**Table 1:** Recombinant influenza A(H6N6) viruses generated in this study

**Strain name^a^**	**HA and NA genes**	**PB2, PB1, PA, NP, MP and NS genes**
rgH6N6-222V/228S	A/swine/Guangdong/K6/2010(H6N6)	A/swine/Guangdong/K6/2010(H6N6)
rgH6N6-222A/228G	A/swine/Guangdong/K6/2010(H6N6)	A/swine/Guangdong/K6/2010(H6N6)
rgH6N6 × PR8-222V/228S	A/swine/Guangdong/K6/2010(H6N6)	A/Puerto Rico/8/1934(H1N1)
rgH6N6 × PR8-222A/228S	A/swine/Guangdong/K6/2010(H6N6)	A/Puerto Rico/8/1934(H1N1)
rgH6N6 × PR8-222V/228G	A/swine/Guangdong/K6/2010(H6N6)	A/Puerto Rico/8/1934(H1N1)
rgH6N6 × PR8-222A/228G	A/swine/Guangdong/K6/2010(H6N6)	A/Puerto Rico/8/1934(H1N1)
rgH6N6 × pdm09-222V/228S	A/swine/Guangdong/K6/2010(H6N6)	A/California/4/2009(H1N1)
rgH6N6 × pdm09-222A/228S	A/swine/Guangdong/K6/2010(H6N6)	A/California/4/2009(H1N1)
rgH6N6 × pdm09-222V/228G	A/swine/Guangdong/K6/2010(H6N6)	A/California/4/2009(H1N1)
rgH6N6 × pdm09-222A/228G	A/swine/Guangdong/K6/2010(H6N6)	A/California/4/2009(H1N1)
rgH3N2 × pdm09	A/swine/Ohio/11SW226/2011(H3N2)	A/California/4/2009(H1N1)

a rg indicates reverse genetics.

**Table 2:** Effect of mutations A222V and G228S of influenza A(H6N6) virus hemagglutinin on binding affinity to erythrocytes from various animals^a^

Virus	Mean hemagglutination titer^b^			
	Turkey	Chicken	Guinea pig	Horse
*Types of erythrocytes*				
rgH6N6 × PR8-222V/228S	32	32	32	32
rgH6N6 × PR8-222A/228S	32	32	16	32
rgH6N6 × PR8-222V/228G	32	64	<2	2
rgH6N6 × PR8-222A/228G	32	64	<2	<2
rgH6N6 × pdm09-222V/228S	32	32	32	16
rgH6N6 × pdm09-222A/228S	32	32	32	32
rgH6N6 × pdm09-222V/228G	32	64	<2	<2
rgH6N6 × pdm09-222A/228G	32	128	<2	2

a rg indicates reverse genetics.

b All virus concentrations were normalized to a hemagglutination titer of 32 in turkey erythrocytes. Each assay was repeated three times, and in each case, the standard deviation was 0.

**Table 3:** Hemagglutination inhibition titers in serum samples from ferrets inoculated with or exposed to animals inoculated with influenza A(H6N6) virus

Virus and experiment group^a^	Time of serum collection^b^	Titer^c^		
*rgH6N6-222V/228S*				
Aerosol transmission study				
Virus inoculated	14 DPI	640	320	1280
Exposure by aerosol	14 DPE	<10	<10	<10
Contact transmission study				
Virus inoculated	14 DPI	640	640	1280
Exposure by direct contact	14 DPE	<10	<10	20

*rgH6N6 × pdm09-222V/228S*				
Aerosol transmission study				
Virus inoculated	14 DPI	1280	1280	1280
Exposure by aerosol	14 DPE	<10	<10	<10
Contact transmission study				
Virus inoculated	14 DPI	1280	1280	640
Exposure by direct contact	14 DPE	640	640	640

a rg indicates reverse genetics.

b Day post exposure, DPE; day post inoculation, DPI.

c Hemagglutination inhibition assays were performed using turkey erythrocytes. The titers were generated from sera collected from three individual ferrets.

**Table 4:** Virus titers in nasal wash fluids of ferrets in aerosol transmission experiment nasally inoculated with influenza A(H6N6) virus

Virus and time (DPI or DPE) of nasal wash fluid collection^a^	Mean virus titer (log_10_EID_50_/mL)^b^		
*rgH6N6-222V/228S*			
2	ND	ND	ND
4	ND	ND	ND
6	1.00	ND	ND
8	1.00	ND	ND

*rgH6N6 × pdm09-222V/228S*			
2	ND	1.00	ND
4	ND	ND	ND
6	ND	ND	ND
8	ND	ND	ND

Abbreviations: day post exposure, DPE; day post inoculation, DPI; egg infectious dose, EID; not detected, ND.

a Data are for virus inoculated ferrets and direct- and aerosol-contact exposure ferrets at various DPI or DPE.

b Virus titers were determined by 50% egg infectious dose (EID_50_) in embryonated chicken eggs. The titers were generated from nasal swabs collected from three individual ferrets.
