# Supplementary material for: Pathogenicity and transmission of a swine influenza A(H6N6) virus
Source: Emerg Microbes Infect. 2017 Apr 12;6(4):e17–. doi: 10.1038/emi.2017.3 (PMC5457681; doi:10.1038/emi.2017.3)
Supplement: Supplementary Information [file emi20173x1.pdf]

Chart number	Chart color	Structure
α2,3 sulfated sialosides		
1		Neu5Aca2-3(6S)Galb1-4(Fuca1-3)GlcNAcb-Sp8
2		Neu5Aca2-3(6S)Galb1-4GlcNAcb-Sp8
3		Neu5Aca2-3Galb1-3(6S)GalNAca-Sp8
4		Neu5Aca2-3Galb1-3(6S)GlcNAc-Sp8
5		Neu5Aca2-3Galb1-4(6S)GlcNAcb-Sp8
α2,3 di-, tri-, and qua-sialosides		
6		Neu5Aca2-3Galb1-3GalNAcb1-4(Neu5Aca2-3)Galb1-4Glcb-Sp0
7		Neu5Aca2-3Galb1-3GalNAcb1-4(Neu5Aca2-8Neu5Aca2-3)Galb1-4Glcb-Sp0
8		Neu5Aca2-3Galb1-3GlcNAcb1-2Mana1-6(GlcNAcb1-4)(Neu5Aca2-3Galb1-3GlcNAcb1-2Mana1-3)Manb1-4GlcNAcb1-4GlcNAc-Sp21
9		Neu5Aca2-3Galb1-3GlcNAcb1-2Mana1-6(Neu5Aca2-3Galb1-3GlcNAcb1-2Mana1-3)Manb1-4GlcNAcb1-4GlcNAc-Sp19
10		Neu5Aca2-3Galb1-3GlcNAcb1-6(Neu5Aca2-3Galb1-4GlcNAcb1-2)Mana1-6(Neu5Aca2-3Galb1-3GlcNAcb1-2Mana1-3)Manb1-4GlcNAcb1-4GlcNAcb-Sp19
11		Neu5Aca2-3Galb1-4GlcNAcb1-2Mana1-6(GlcNAcb1-4)(Neu5Aca2-3Galb1-4GlcNAcb1-2Mana1-3)Manb1-4GlcNAcb1-4GlcNAcb-Sp21
12		Neu5Aca2-3Galb1-4GlcNAcb1-2Mana1-6(Neu5Aca2-3Galb1-4GlcNAcb1-2Mana1-3)Manb1-4GlcNAcb1-4(Fuca1-6)GlcNAcb-Sp24
13		Neu5Aca2-3Galb1-4GlcNAcb1-2Mana1-6(Neu5Aca2-3Galb1-4GlcNAcb1-2Mana1-3)Manb1-4GlcNAcb1-4GlcNAcb-Sp12
14		Neu5Aca2-3Galb1-4GlcNAcb1-3Galb1-4GlcNAcb1-2Mana1-6(Neu5Aca2-3Galb1-4GlcNAcb1-3Galb1-4GlcNAcb1-2Mana1-3)Manb1-4GlcNAcb1-4GlcNAcb-Sp12
15		Neu5Aca2-3Galb1-4GlcNAcb1-3Galb1-4GlcNAcb1-3Galb1-4GlcNAcb1-2Mana1-6(Neu5Aca2-3Galb1-4GlcNAcb1-3Galb1-4GlcNAcb1-3Galb1-4GlcNAcb1-2Mana1-3)Manb1-4GlcNAcb1-4GlcNAcb-Sp12
16		Neu5Aca2-3Galb1-4GlcNAcb1-3Galb1-4GlcNAcb1-6(Neu5Aca2-3Galb1-4GlcNAcb1-3Galb1-4GlcNAcb1-3)GalNAca-Sp14
17		Neu5Aca2-3Galb1-4GlcNAcb1-6(Neu5Aca2-3Galb1-3)GalNAca-Sp14
18		Neu5Aca2-3Galb1-4GlcNAcb1-6(Neu5Aca2-3Galb1-4GlcNAcb1-3)GalNAca-Sp14
19		Neu5Gca2-3Galb1-4GlcNAcb1-3Galb1-4GlcNAcb1-2Mana1-6(Neu5Gca2-3Galb1-4GlcNAcb1-3Galb1-4GlcNAcb1-2Mana1-3)Manb1-4GlcNAcb1-4GlcNAcb-Sp24
20		Neu5Aca2-3Galb1-4GlcNAcb1-4Mana1-6(GlcNAcb1-4)(Neu5Aca2-3Galb1-4GlcNAcb1-4(Neu5Aca2-3Galb1-4GlcNAcb1-2)Mana1-3)Manb1-4GlcNAcb1-4GlcNAcb-Sp21
21		Neu5Aca2-3Galb1-4GlcNAcb1-6(Neu5Aca2-3Galb1-4GlcNAcb1-2)Mana1-6(GlcNAcb1-4)(Neu5Aca2-3Galb1-4GlcNAcb1-2Mana1-3)Manb1-4GlcNAcb1-4GlcNAcb-Sp21
22		Neu5Aca2-3Galb1-4GlcNAcb1-6(Neu5Aca2-3Galb1-4GlcNAcb1-2)Mana1-6(GlcNAcb1-4)(Neu5Aca2-3Galb1-4GlcNAcb1-4(Neu5Aca2-3Galb1-4GlcNAcb1-2)Mana1-3)Manb1-4GlcNAcb1-4GlcNAcb-Sp21
α2,3 linear sialosides		
23		Neu5Aca2-3Galb1-3GalNAca-Sp8
24		Neu5Aca2-3Galb1-3GalNAcb1-3Gala1-4Galb1-4Glcb-Sp0
25		Neu5Aca2-3Galb1-3GlcNAcb1-2Mana-Sp0
26		Neu5Aca2-3Galb1-3GlcNAcb1-3Galb1-3GlcNAcb-Sp0
27		Neu5Aca2-3Galb1-3GlcNAcb1-3Galb1-4GlcNAcb-Sp0
28		Neu5Aca2-3Galb1-3GlcNAcb1-3GalNAca-Sp14
29		Neu5Aca2-3Galb1-3GlcNAcb1-4Galb1-4Glcb-Sp0
30		Neu5Aca2-3Galb1-3GlcNAcb1-6GalNAca-Sp14

31		Neu5Aca2-3Galb1-3GlcNAcb-Sp0
32		Neu5Aca2-3Galb1-3GlcNAcb-Sp8
33		Neu5Aca2-3Galb1-4GlcNAcb1-2Mana-Sp0
34		Neu5Aca2-3Galb1-4GlcNAcb1-3Galb1-3GlcNAcb-Sp0
35		Neu5Aca2-3Galb1-4GlcNAcb1-3Galb1-4GlcNAcb1-3Galb1-4GlcNAcb-Sp0
36		Neu5Aca2-3Galb1-4GlcNAcb1-3Galb1-4GlcNAcb1-3GalNAca-Sp14
37		Neu5Aca2-3Galb1-4GlcNAcb1-3Galb1-4GlcNAcb1-6(Galb1-3)GalNAca-Sp14
38		Neu5Aca2-3Galb1-4GlcNAcb1-3Galb1-4GlcNAcb-Sp0
39		Neu5Aca2-3Galb1-4GlcNAcb1-3Galb-Sp8
40		Neu5Aca2-3Galb1-4GlcNAcb1-3GalNAc-Sp14
41		Neu5Aca2-3Galb1-4GlcNAcb1-6GalNAca-Sp14
42		Neu5Aca2-3Galb1-4GlcNAcb-Sp0
43		Neu5Aca2-3Galb1-4GlcNAcb-Sp8
44		Neu5Aca2-3Galb-Sp8
45		Neu5Aca2-3GalNAca-Sp8
46		Neu5Aca2-3GalNAcb1-4GlcNAcb-Sp0
47		Neu5Gca2-3Galb1-3GlcNAcb-Sp0
48		Neu5Gca2-3Galb1-4Glcb-Sp0
49		Neu5Gca2-3Galb1-4GlcNAcb-Sp0
α2,3 Fucosylated sialosides		
50		Neu5Aca2-3Galb1-3(Fuca1-4)GlcNAcb1-3Galb1-3(Fuca1-4)GlcNAcb-Sp0
51		Neu5Aca2-3Galb1-3(Fuca1-4)GlcNAcb1-3Galb1-4(Fuca1-3)GlcNAcb-Sp0
52		Neu5Aca2-3Galb1-3(Fuca1-4)GlcNAcb-Sp8
53		Neu5Aca2-3Galb1-3GalNAca-Sp14
54		Neu5Aca2-3Galb1-4(Fuca1-3)(6S)GlcNAcb-Sp8
55		Neu5Aca2-3Galb1-4(Fuca1-3)GlcNAcb1-2Mana-Sp0
56		Neu5Aca2-3Galb1-4(Fuca1-3)GlcNAcb1-3Galb1-4(Fuca1-3)GlcNAcb1-3Galb1-4(Fuca1-3)GlcNAcb-Sp0
57		Neu5Aca2-3Galb1-4(Fuca1-3)GlcNAcb1-3Galb1-4GlcNAcb-Sp8
58		Neu5Aca2-3Galb1-4(Fuca1-3)GlcNAcb1-3Galb-Sp8
59		Neu5Aca2-3Galb1-4(Fuca1-3)GlcNAcb1-3GalNAca-Sp14
60		Neu5Aca2-3Galb1-4(Fuca1-3)GlcNAcb1-6(Galb1-3)GalNAca-Sp14
61		Neu5Aca2-3Galb1-4(Fuca1-3)GlcNAcb1-6(Neu5Aca2-3Galb1-3)GalNAc-Sp14
62		Neu5Aca2-3Galb1-4(Fuca1-3)GlcNAcb-Sp0
63		Neu5Aca2-3Galb1-4(Fuca1-3)GlcNAcb-Sp8
64		Neu5Aca2-3Galb1-4(Neu5Aca2-3Galb1-3)GlcNAcb-Sp8

65		Neu5Aca2-3Galb1-4Glcb-Sp0
66		Neu5Aca2-3Galb1-4Glcb-Sp8
67		Neu5Aca2-3Galb1-4GlcNAcb1-3Galb1-4(Fuca1-3)GlcNAcb-Sp0
68		Neu5Gca2-3Galb1-3(Fuca1-4)GlcNAcb-Sp0
69		Neu5Gca2-3Galb1-4(Fuca1-3)GlcNAcb-Sp0
α2,3 internal sialosides		
70		Galb1-3GalNAcb1-4(Neu5Aca2-8Neu5Aca2-3)Galb1-4Glcb-Sp0
71		Galb1-3GalNAcb1-4(Neu5Aca2-8Neu5Aca2-8Neu5Aca2-3)Galb1-4Glcb-Sp21
72		GalNAcb1-4(Neu5Aca2-3)Galb1-4Glcb-Sp0
73		GalNAcb1-4(Neu5Aca2-3)Galb1-4GlcNAcb1-3GalNAca-Sp14
74		GalNAcb1-4(Neu5Aca2-3)Galb1-4GlcNAcb-Sp0
75		GalNAcb1-4(Neu5Aca2-3)Galb1-4GlcNAcb-Sp8
76		GalNAcb1-4(Neu5Aca2-8Neu5Aca2-3)Galb1-4Glcb-Sp0
77		GalNAcb1-4(Neu5Aca2-8Neu5Aca2-8Neu5Aca2-3)Galb1-4Glcb-Sp0
78		GalNAcb1-4(Neu5Aca2-8Neu5Aca2-8Neu5Aca2-8Neu5Aca2-3)Galb1-4Glcb-Sp0
79		GlcNAcb1-6(Neu5Aca2-3Galb1-3)GalNAca-Sp14
80		Neu5Aca2-3Galb1-4GlcNAcb1-6(Galb1-3)GalNAca-Sp14
81		Neu5Aca2-8Neu5Aca2-3Galb1-3GalNAcb1-4(Neu5Aca2-3)Galb1-4Glc-Sp21
82		Neu5Aca2-8Neu5Aca2-3Galb1-3GalNAcb1-4(Neu5Aca2-8Neu5Aca2-3)Galb1-4Glcb-Sp0
83		Neu5Aca2-8Neu5Aca2-3Galb1-4Glcb-Sp0
84		Neu5Aca2-8Neu5Aca2-3Galb1-4GlcNAc-Sp0
85		Neu5Aca2-8Neu5Aca2-8Neu5Aca2-3Galb1-4Glcb-Sp0
86		Neu5Aca2-8Neu5Gca2-3Galb1-4GlcNAc-Sp0
87		Neu5Gca2-8Neu5Aca2-3Galb1-4GlcNAc-Sp0
88		Neu5Gca2-8Neu5Gca2-3Galb1-4GlcNAcb1-3Galb1-4GlcNAc-Sp0
89		Neu5Gca2-8Neu5Gca2-3Galb1-4GlcNAc-Sp0
90		Fuca1-2Galb1-3GalNAcb1-4(Neu5Aca2-3)Galb1-4Glcb-Sp0
91		Fuca1-2Galb1-3GalNAcb1-4(Neu5Aca2-3)Galb1-4Glcb-Sp9
92		Galb1-3GalNAcb1-4(Neu5Aca2-3)Galb1-4Glcb-Sp0
α2,3 α2,6 sialosides		
93		Neu5Aca2-6Galb1-4GlcNAcb1-2Mana1-6(Neu5Aca2-3Galb1-4GlcNAcb1-2Mana1-3)Manb1-4GlcNAcb1-4GlcNAcb-Sp12
94		Neu5Aca2-6(Neu5Aca2-3)GalNAca-Sp8
95		Neu5Aca2-6(Neu5Aca2-3Galb1-3)GalNAca-Sp14
96		Neu5Aca2-6(Neu5Aca2-3Galb1-3)GalNAca-Sp8

97		Neu5Aca2-3Galb1-4GlcNAcb1-2Mana1-6(Neu5Aca2-6Galb1-4GlcNAcb1-2Mana1-3)Manb1-4GlcNAcb1-4GlcNAcb-Sp12
98		Galb1-4(Fuca1-3)GlcNAcb1-6(Neu5Aca2-6(Neu5Aca2-3Galb1-3)GlcNAcb1-3)Galb1-4Glc-Sp21

α2,6 sialosides in the same order to α2,3

99		Neu5Aca2-6Galb1-4(6S)GlcNAcb-Sp8
100		Neu5Aca2-6GalNAcb1-4(6S)GlcNAcb-Sp8
101		Neu5Aca2-6Galb1-4 GlcNAcb1-6(Neu5Aca2-6Galb1-4GlcNAcb1-3)GalNAca-Sp14
102		Neu5Aca2-6Galb1-4GlcNAcb1-2Mana1-6(GlcNAcb1-4)(Neu5Aca2-6Galb1-4GlcNAcb1-2Mana1-3)Manb1-4GlcNAcb1-4GlcNAcb-Sp21
103		Neu5Aca2-6Galb1-4GlcNAcb1-2Mana1-6(Neu5Aca2-6Galb1-4GlcNAcb1-2Mana1-3)Manb1-4GlcNAcb1-4(Fuca1-6)GlcNAcb-Sp24
104		Neu5Aca2-6Galb1-4GlcNAcb1-2Mana1-6(Neu5Aca2-6Galb1-4GlcNAcb1-2Mana1-3)Manb1-4GlcNAcb1-4GlcNAcb-Sp12
105		Neu5Aca2-6Galb1-4GlcNAcb1-2Mana1-6(Neu5Aca2-6Galb1-4GlcNAcb1-2Mana1-3)Manb1-4GlcNAcb1-4GlcNAcb-Sp13
106		Neu5Aca2-6Galb1-4GlcNAcb1-2Mana1-6(Neu5Aca2-6Galb1-4GlcNAcb1-2Man-a1-3)Manb1-4GlcNAcb1-4GlcNAcb-Sp21
107		Neu5Aca2-6Galb1-4GlcNAcb1-2Mana1-6(Neu5Aca2-6Galb1-4GlcNAcb1-2Mana1-3)Manb1-4GlcNAcb1-4GlcNAcb-Sp24
108		Neu5Aca2-6Galb1-4GlcNAcb1-3Galb1-4GlcNAcb1-2Mana1-6(Neu5Aca2-6Galb1-4GlcNAcb1-3Galb1-4GlcNAcb1-2Mana1-3)Manb1-4GlcNAcb1-4GlcNAcb-Sp12
109		Neu5Aca2-6Galb1-4GlcNAcb1-3Galb1-4GlcNAcb1-3Galb1-4GlcNAcb1-2Mana1-6(Neu5Aca2-6Galb1-4GlcNAcb1-3Galb1-4GlcNAcb1-3Galb1-4GlcNAcb1-2Mana1-3)Manb1-4GlcNAcb1-4GlcNAcb-Sp12
110		Neu5Aca2-6Galb1-4GlcNAcb1-3Galb1-4GlcNAcb1-6(Neu5Aca2-6Galb1-4GlcNAcb1-3Galb1-4GlcNAcb1-3)GalNAca-Sp14
111		Neu5Aca2-6Galb1-4GlcNAcb1-4Mana1-6(GlcNAcb1-4)(Neu5Aca2-6Galb1-4GlcNAcb1-4(Neu5Aca2-6Galb1-4GlcNAcb1-2)Mana1-3)Manb1-4GlcNAcb1-4GlcNAcb-Sp21
112		Neu5Aca2-6Galb1-4GlcNAcb1-6(Neu5Aca2-6Galb1-4GlcNAcb1-2)Mana1-6(GlcNAcb1-4)(Neu5Aca2-6Galb1-4GlcNAcb1-2Mana1-3)Manb1-4GlcNAcb1-4GlcNAcb-Sp21
113		Neu5Aca2-6Galb1-4GlcNAcb1-6(Neu5Aca2-6Galb1-4GlcNAcb1-2)Mana1-6(GlcNAcb1-4)(Neu5Aca2-6Galb1-4GlcNAcb1-4(Neu5Aca2-6Galb1-4GlcNAcb1-2)Mana1-3)Manb1-4GlcNAcb1-4GlcNAcb-Sp21
114		Neu5Aca2-6Galb1-4Glcb-Sp0
115		Neu5Aca2-6Galb1-4Glcb-Sp8
116		Neu5Aca2-6Galb1-4GlcNAcb1-2Mana1-3Manb1-4GlcNAcb1-4GlcNAc-Sp12
117		Neu5Aca2-6Galb1-4GlcNAcb1-2Mana1-6Manb1-4GlcNAcb1-4GlcNAc-Sp12
118		Neu5Aca2-6Galb1-4GlcNAcb1-2Man-Sp0
119		Neu5Aca2-6Galb1-4GlcNAcb1-3Galb1-3GlcNAcb-Sp0
120		Neu5Aca2-6Galb1-4GlcNAcb1-3Galb1-4GlcNAcb1-3Galb1-4GlcNAcb-Sp0
121		Neu5Aca2-6Galb1-4GlcNAcb1-3Galb1-4GlcNAcb1-3GalNAca-Sp14
122		Neu5Aca2-6Galb1-4GlcNAcb1-3Galb1-4GlcNAcb-Sp0
123		Neu5Aca2-6Galb1-4GlcNAcb1-3GalNAc-Sp14
124		Neu5Aca2-6Galb1-4GlcNAcb1-6GalNAca-Sp14
125		Neu5Aca2-6Galb1-4GlcNAcb-Sp0
126		Neu5Aca2-6Galb1-4GlcNAcb-Sp8
127		Neu5Aca2-6Galb-Sp8
128		Neu5Aca2-6GalNAca-Sp8
129		Neu5Aca2-6GalNAcb1-4GlcNAcb-Sp0
130		Neu5Aca2-6GlcNAcb1-4GlcNAcb1-4GlcNAc-Sp21
131		Neu5Aca2-6GlcNAcb1-4GlcNAc-Sp21

132		Neu5Acb2-6Galb1-4GlcNAcb-Sp8
133		Neu5Acb2-6GalNAca-Sp8
134		Neu5Gca2-6Galb1-4GlcNAcb-Sp0
135		Neu5Gca2-6GalNAca-Sp0
136		Neu5Aca2-6Galb1-4GlcNAcb1-3Galb1-4(Fuca1-3)GlcNAcb1-3Galb1-4(Fuca1-3)GlcNAcb-Sp0
137		Neu5Aca2-6Galb1-4GlcNAcb1-6(Fuca1-2Galb1-3GlcNAcb1-3)Galb1-4Glc-Sp21
138		Neu5Aca2-6Galb1-4GlcNAcb1-6(Fuca1-2Galb1-4(Fuca1-3)GlcNAcb1-3)Galb1-4Glc-Sp21
139		Galb1-4GlcNAcb1-2Mana1-6(Neu5Aca2-6Galb1-4GlcNAcb1-2Mana1-3)Manb1-4GlcNAcb1-4GlcNAcb-Sp12
140		Galb1-4GlcNAcb1-6(Neu5Aca2-6Galb1-3GlcNAcb1-3)Galb1-4Glc-Sp21
141		GlcNAcb1-2Mana1-6(Neu5Aca2-6Galb1-4GlcNAcb1-2Mana1-3)Manb1-4GlcNAcb1-4GlcNAcb-Sp12
142		Mana1-6(Neu5Aca2-6Galb1-4GlcNAcb1-2Mana1-3)Manb1-4GlcNAcb1-4GlcNAc-Sp12
143		Neu5Aca2-6(Galb1-3)GalNAca-Sp14
144		Neu5Aca2-6(Galb1-3)GalNAca-Sp8
145		Neu5Aca2-6(Galb1-3)GlcNAcb1-4Galb1-4Glcb-Sp10
146		Neu5Aca2-6Galb1-4GlcNAcb1-2Mana1-6(Galb1-4GlcNAcb1-2Mana1-3)Manb1-4GlcNAcb1-4GlcNAcb-Sp12
147		Neu5Aca2-6Galb1-4GlcNAcb1-2Mana1-6(GlcNAcb1-2Mana1-3)Manb1-4GlcNAcb1-4GlcNAcb-Sp12
148		Neu5Aca2-6Galb1-4GlcNAcb1-2Mana1-6(Mana1-3)Manb1-4GlcNAcb1-4GlcNAc-Sp12
149		Neu5Aca2-6Galb1-4GlcNAcb1-3Galb1-4GlcNAcb1-6(Galb1-3)GalNAca-Sp14
150		Neu5Aca2-6Galb1-4GlcNAcb1-6(Galb1-3)GalNAca-Sp14
151		Neu5Aca2-6Galb1-4GlcNAcb1-6(Galb1-3GlcNAcb1-3)Galb1-4Glcb-Sp21
152		Neu5Acb2-6(Galb1-3)GalNAca-Sp8
153		Neu5Gca2-8Neu5Gca2-6Galb1-4GlcNAc-Sp0

The table is color-coded according to text Figure 4 and glycans are consistent with Figure 4.
Key: Neu5Ac, sialic acid; OSO3, sulfate; Gal, galactose; Fuc, fucose; GalNAc, *N*-acetyl-d-galactosamine; Glc, glucose; Man, mannose; SP0, -$CH_2CH_2NH_2$; SP8, -$CH_2CH_2CH_2NH_2$; Sp9, -$CH_2CH_2CH_2CH_2CH_2NH_2$; Sp10, -$NHCOCH_2NH$; SP12, Asparagine; Sp13, glycine; SP14, Threonine; Sp19, EN or NK; Sp21, -$N(CH_3)$-O-$(CH_2)_2$-NH_2; Sp24, KVANKT.

Supplementary Table S1 Sialic acid–linked glycans represented on the glycan microarray
